# Out-of-hospital resuscitation of a 3 month old boy presenting with recurrent ventricular fibrillation cardiac arrest: a case report

**DOI:** 10.1186/s13049-021-00871-9

**Published:** 2021-04-13

**Authors:** Peter Kingsley, Jonathan Merefield, Robert G. Walker, Fred W. Chapman, Mark Faulkner

**Affiliations:** 1 London Ambulance Service NHS Trust, London Ambulance Service, 220 Waterloo Road, London, SE1 8SD UK; 2 Stryker Emergency Care, 11811 Willows Road NE, Redmond, WA 98052 USA

**Keywords:** Paediatric, Resuscitation, Cardiac arrest, Paramedic, Advanced practice

## Abstract

A 3 month old boy, with no known health conditions, suffered a sudden collapse at home. On first EMS arrival, ventricular fibrillation (VF) cardiac arrest was identified and resuscitation following UK national guidelines was initiated. He remained in cardiac arrest for over 25 min, during which he received 10 defibrillation shocks, each effective, but with VF reoccurring within a few seconds of each of the first 9. A return of spontaneous circulation (ROSC) was achieved after the 10th shock. The resuscitation was conducted fully in his home, with the early involvement of Advanced Paramedic Practitioners specialising in critical care (APP- CC). Throughout his resuscitation, there remained a strong focus on delivering quality resuscitation in situ, rather than a ‘load and go’ approach that would have resulted in very early conveyance to hospital with on-going CPR.

The patient was subsequently discharged home and is making an excellent recovery. The arrest was later determined to have been caused by a primary arrhythmia as a result of a previously unidentified non-obstructive variant hypertrophic cardiomyopathy.

We present data downloaded from the defibrillator used during the resuscitation that illustrates clearly the recurrent nature of his fibrillation.

## Background

National and regional registry data indicates an incidence of 47–72 cases per 100,000 years [1, 2] of sudden cardiac arrest in infancy. Within this small group of patients, the occurrence of shockable ventricular dysrhythmias is estimated to be below 10%, reflecting a higher likelihood for young children to suffer a primary non-cardiac cause for their collapse [1, 2].

These findings are reflected in data published by the London Ambulance Service NHS Trust (LAS). Serving a population of approximately 9 million people, in the year 2018–2019 LAS clinicians delivered CPR to 4004 patients presenting with out of hospital cardiac arrest (OHCA). Of these, only 54 were for patients under one year old, with none in a shockable rhythm on EMS arrival and only 6 (11%) surviving to hospital discharge [3].

The overall low numbers of out-of-hospital cardiac arrests, together with the obvious ethical restrictions on clinical trials in children, has resulted in those charged with authoring national guidelines relying predominantly on findings drawn from case reports, retrospective registry studies, animal modelling and studies exploring resuscitation in the adult population [4–7].

This results in a lack of high-quality evidence on which to make recommendations on many fundamental aspects of a resuscitation. For example, there is significant uncertainty as to; the optimal level of energy required when attempting defibrillation; how best to manage the paediatric airway; how long to stay on scene vs when to leave for hospital with resuscitation on-going (load and go) [4, 6–8].

Similarly, guidance around the use of antiarrhythmic drugs and the optimal dosing of intra-arrest adrenaline have been largely drawn from studies conducted on animals and adult populations, with results of recent large-scale trials arguably equivocal at best [5].

Given the very low incidence of out of hospital cardiac arrests in the infant population, the likelihood is that most EMS crews will work for many months or even years without finding themselves having to undertake such a resuscitation. In UK practice it is standard practice for EMS providers to mandate that their clinicians conduct OHCA resuscitations with close adherence to UK national resuscitation guidelines [4].

Occurring within the Greater London area, the incident described in this case report was managed by the LAS. Upon receipt of an OHCA call, it is LAS policy to immediately dispatch a minimum of 5 clinicians to the scene. In the majority of incidents, this response consists of a combination of ALS provider paramedics working with Emergency Ambulance Crew (EAC) colleagues. In addition, a message is sent via the Good Sam web application seeking the assistance of local volunteer responders and a further message is sent to the police, all of whom are trained in CPR and many carry an AED in their patrol car. Management of the response is provided by Emergency Medical Dispatchers (EMD) working in one of two Emergency Operations Centres (EOC). Depending on the nature of the call, the EMD also has the option to allocate an Incident Response Officer (IRO) and/ or a Clinical Team Manager (CTM). In addition, such calls are brought to the attention of the Advanced Paramedic Practitioner – Critical Care (APP-CC) who will provide clinical oversight and management of the incident, either remotely from the operations centre, or through dispatch of an APP-CC colleague to attend the scene.

Working as a single responder on a fast response car, the LAS APP-CC clinicians are tasked to augment and enhance the clinical response provided in high acuity medical and trauma calls across the city. Educated to master’s degree level, and working within a tightly governed scheme, they are able to deliver a range of enhanced patient interventions, advanced level decision making and much greater exposure (and so familiarity) with rare cases such as this [9]. At any time there are a maximum of 5 operational APP-CCs and a further one working in the EOC to respond to incidents across the Greater London area.

## Case presentation

The patient was a 3 month old male of African American descent. He was reported to be a healthy child with no diagnosed medical conditions. Born at 38 weeks with a relatively low birth weight (2.5kgs) he had developed along expected norms and had received standard UK immunisations for his age. He was 5.7kgs and had a body length of 58cms at the time of this sudden illness. In the days leading up to his collapse, his mother had some concerns around what she described as ‘reflux’ type symptoms, including some mild infrequent vomiting and restless nights. She also reports her son being ‘clammy’ with cool but sweaty skin. At the time she did not find these issues overly concerning and managed them at home and did not seek medical advice.

On the morning of his collapse he woke around 05:00 and was fed by his mother. She then put him in a bassinet in a bedroom on the first floor of their house. She recalls that he was particularly restless and agitated and took some time to settle back to sleep. Around an hour later she noticed he had become extremely restless and agitated. She picked him up and found him to be ‘floppy’ and ‘flaccid’. He then began twitching and his breathing became abnormal. Within a few seconds his lips had become blue, he was ‘gasping’ and ‘no longer awake’.

The mother called 999 at 06.56 and was connected to a LAS Emergency Call Handler. During the course of the emergency call a breathing assessment was carried out, which correctly identified abnormal and likely ineffective breathing. As a result, a Category 1 (highest priority) response was generated. The mother and father delivered rescue breaths to their son whilst on the phone to the emergency services.

Two double crewed ambulances and a paramedic working on a fast response care were immediately dispatched. In addition, an IRO was allocated to the call and the incident was immediately brought to the attention of the APP-CC who dispatched a colleague to attend.

The first LAS crew arrived at the patient within 6 min of the 999 call. At this time the infant had been placed on his back, on the floor in the upstairs bedroom. There was no CPR in progress. The crew noted the patient was centrally and peripherally cyanosed with an agonal respiratory rate of approximately 4/min. Cardiac and respiratory arrest was identified and basic life support resuscitation initiated. Paediatric-specific defibrillator pads were placed in the anterior/ posterior chest position and connected to an automatic external defibrillator (LIFEPAK 1000, Stryker Emergency Care, Redmond, WA, USA). The AED advised a shock following its initial rhythm analysis, and the first shock was delivered at 07.05. Chest compressions were initiated after the shock and followed a ratio of 15 compressions to 2 ventilations throughout. CPR was paused briefly at two-minute intervals to allow for cardiac rhythm analysis. At each of the first ten analysis points VF was identified and a shock delivered.

Advanced life support was initiated with the placement of an intraosseous needle in the right proximal tibia and a supraglottic airway (iGEL size 1.5). The infant received a single bolus dose of 30 mg Amiodarone after the 3rd shock and 60mcg Adrenaline (0.1 mg/kg with an estimated weight of 6kgs) following the 3rd, 5th and 7th shocks.

Following the 7th shock contact was made with the APP-CC in the EOC. This allowed one of the attending paramedics to discuss the situation with a senior clinician and obtain advice and guidance and to ‘sense check’ the interventions delivered thus far and plan how to proceed. The advice included to omit the next dose of adrenaline in order to ascertain whether excessive adrenergic stimulation was prolonging the arrhythmia. An APP-CC arrived with the patient between the 9th and 10th shocks. He initiated a brief post-shock pause following the 10th shock and almost immediately saw what appeared to be organised cardiac activity on the monitor. Within 10–15 s the rate increased and the complexes began to narrow. An associated rapid rise in end tidal carbon dioxide (EtCO2) reading was also noted. At 30 s post shock the rate and morphology of the ECG had further normalized, however there remained no palpable pulses. Two further cycles of 15:2 CPR were delivered. Following this, a central pulse was felt and point-of-care ultrasound confirmed organised cardiac activity.

Immediately following ROSC, the infant was allowed to rest in situ for 4–5 min to allow a period of minimal handling and stimulation. Having displayed agonal breathing throughout the resuscitation, he then quickly established a more effective rate and depth of spontaneous ventilations through the iGel. Three minutes post ROSC he had a non-invasive systolic blood pressure reading of 80 mmHg and an EtCO2 reading of 5.0kpa.

The infant was then carried downstairs to the waiting ambulance and conveyed to the nearest paediatric Emergency Department (ED). During the 10-min journey he started to cough on the iGel and so this was removed. He was then able to maintain his own airway. On arrival at the ED his eyes were open, he was crying and moving all four limbs in response to gentle tactile stimulation.

Post-handover to the hospital resuscitation team he suffered a further 2 cardiac arrests, from which he was successfully resuscitated. Immediately following the resuscitation all of the EMS clinicians involved were brought together for a ‘hot debrief’ lead by the APP-CC. As part of this process the data from the AED was downloaded to a laptop computer. Primarily intended to allow for a period of mental decompression, the debrief also enabled discussion and reflection on aspects of the resuscitation. Data sourced from the AED is discussed in the next section.

The infant was later transferred to a specialist tertiary centre where a diagnosis of non-obstructive variant hypertrophic cardiomyopathy was made. The aetiology remains uncertain. Following a prolonged and complex period of in-hospital management and recovery, he was discharged home with frequent follow-up and onward care with the specialist teams. Neurological imaging and examination identified evidence of diffuse hypoxic brain injury. On assessment prior to discharge from hospital he demonstrated a suboptimal score of 61 on the Hammersmith Infant Neurological Assessment [10] (at 6 months of age) and delays in his gross motor skills based on the Bayley III Scales of Infant and Toddler Development [11]. His development was in the typical range in the other domains of development i.e. cognition, language and fine motor. He has since celebrated his 1st birthday at home with his parents and continues to make progress in his development.

### AED data review

Post incident, the AED data was downloaded and analysed using CODE-STAT 11 data review software. Review showed a compression to ventilation ratio of 15:2 and average compression rate of 105/min (range 100–120). In total, the AED analysed the ECG rhythm 16 times over the course of the resuscitation and post-ROSC care and advised shocks on 10 occasions. All of the shock advisory decisions were confirmed to be accurate.

**Fig. 1:**
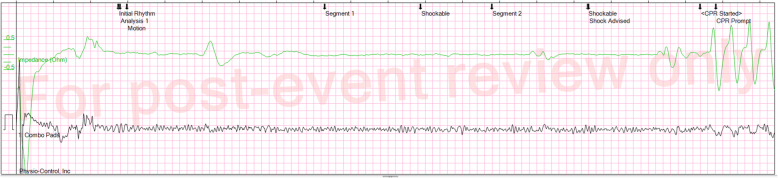
Initial Presenting Rhythm

**Fig. 2:**
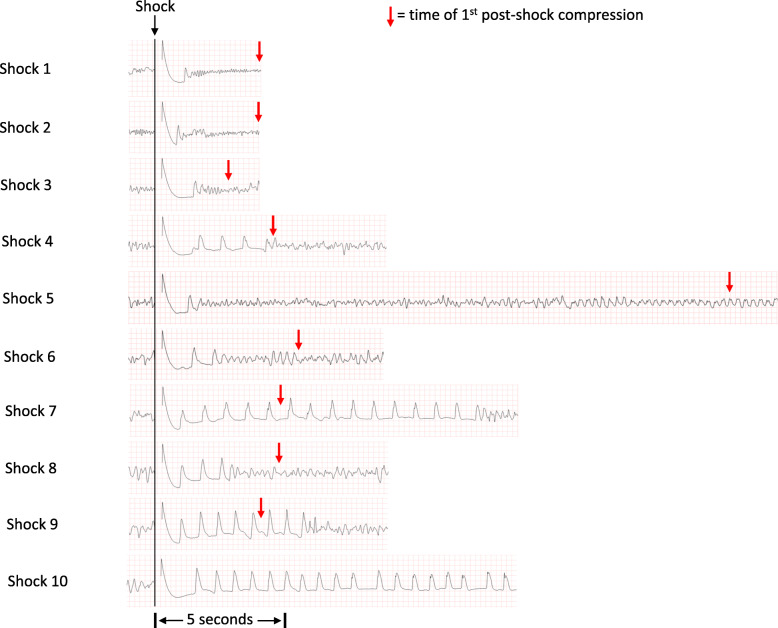
Cardiac rhythm at each analysis point and time chest compressions recommenced

Figure 1 shows the initial presenting rhythm. Figure 2 shows ECG strips surrounding each of the defibrillation shocks, culminating in a return of circulation after shock number 10. A red arrow indicates the point at which chest compressions recommenced.

The AED was configured to deliver 360 joules of biphasic energy, however the use of paediatric specific electrodes had the effect of attenuating the delivered energy by a factor of 4, resulting in an actual energy level of 90 joules.

## Discussion

This case report describes the successful resuscitation of a very young patient who presented with a lethal cardiac dysrhythmia in a community setting. A very rare event and one from which very few infants survive. The data presented illustrates how the resuscitation of this infant broadly followed national and international guidelines however, considered clinical judgement and reasoning resulted in it deviating in a number of key areas:

EMS are advised to consider conveying all paediatric patients who remain in arrest after an initial period of resuscitation to the closest suitable receiving hospital. However, the optimal timings for this remain uncertain and are certainly case specific [4, 6, 7]. Repeated studies [4, 6–8] have shown that the quality of the resuscitation diminishes significantly when the patient is moved. In this case, to achieve this would have required quality CPR to be conducted whilst carrying the infant downstairs, on to an ambulance and then in a moving vehicle on route to hospital. In conjunction with the APP-CC in the EOC, a decision was made to remain on scene to optimise delivery of high-quality resuscitation until the arrival of more senior support. The presence of agonal respirations throughout the resuscitation and the remarkably positive final outcome speak to highly effective cerebral flow being maintained throughout this resuscitation.

The decision to omit a dose of adrenaline reflects concern that excessive sympathetic stimulation may prolong arrhythmias in primary arrhythmogenic cardiac arrests. Evidence to support this is very limited, however so too is evidence to support a 3–5 min administration during primary arrhythmogenic cardiac arrests in infancy. It is interesting to note that in this case, ROSC was achieved approximately seven minutes after the last dose of adrenaline.

Post shock pauses are not recommended in guidelines for very good reason [6]. Any pause in compressions will lead to a rapid fall in coronary and cerebral blood flow. However, such a pause is a useful and informative intervention for an experienced resuscitation practitioner to undertake. They allow for a better understanding of the efficacy of the defibrillation shock, separating recurrent from true refractory VF (mandating a change in strategy). Furthermore, they allow examination of whether it is the compressions themselves that are irritating the myocardium and prolonging the lethal arrhythmia. Close examination of the data from the AED in this case reveals an interesting association between initiation of chest compressions and a return of fibrillation following a number of the shocks. It was only after a carefully considered and managed post shock pause that ROSC was achieved.

Guidelines recommend a dose of 2 to 4 J/kg for paediatric manual defibrillation, or use of an AED with a paediatric attenuator, and additionally advise that AEDs that deliver adult energy dosages are acceptable if a paediatric attenuator is not available [6]. At 5.7 kg and an energy level of 90 J, this child received a dose of ~ 16 J/kg. In a prior published case report of an infant treated with AED defibrillation with a paediatric energy attenuator [12], Bar-Cohen et al. describe a 6.3 kg infant treated with an energy level of 50 J. The authors report that while there was marked bradycardia for nearly 60 s after the shock, there was no evidence of significant injury. In the present case, an organized rhythm with a rate > 60 was observed immediately after shock delivery, until the moment of refibrillation (Fig. 2). As in the case reported by Bar-Cohen et al., no evidence of significant injury was observed.

The AED identified a shockable rhythm on initial patient connection and after each of the next 9 CPR cycles. From a resuscitation process and clinical decision-making perspective, this sequence would appear to represent “refractory” VF, potentially indicating that additional defibrillation energy or an alternative shock vector might be warranted. However, review of the recorded data reveals that the VF encountered after the initial shock was instead 9 consecutive episodes of recurrent VF. Each of the first 9 shocks terminated VF for between 1 and 13 s, allowing a transient organized rhythm to be established. The need for repeated shocks in this resuscitation event was thus not due to a failure of the electrophysiologic process of defibrillation, but rather was presumably a consequence of a highly arrhythmogenic myocardial substrate secondary to the undiagnosed hypertrophic cardiomyopathy.

## Conclusion

Sudden cardiac arrest is an extremely rare event in the infant population and shockable cardiac rhythm on EMS arrival even rarer. As a result, the optimal clinical management of such patients is uncertain, particularly when standard treatments have failed to restore organised cardiac output. It is for this reason that such resuscitations should be reported and shared as they perform an important role in informing the evidence base.

We suggest that the decision of the attending clinicians to remain on-scene ensured that they were able to remain focussed on delivering high-quality essential life support. This, together with the early support provided by specialist practitioners, ensured that the infant’s care was refined and individualised to his clinical needs and resulted in a very favourable outcome.

## Data Availability

All data generated or analysed during this study are included in this published article.

## Abbreviations

AED: Automated External Defibrillator
ALS: Advanced Life Support
APP-CC: Advanced Paramedic Practitioner – Critical Care
CTM: Clinical Team Manager
EAC: Emergency Ambulance Crew
EMD: Emergency Medical Dispatcher
EOC: Emergency Operations Centre
ETCO2: End Tidal Carbon Dioxide
IRO: Incident Response Officer
LAS: London Ambulance Service NHS Trust
ROSC: Return of Spontaneous Circulation
VF: Ventricular Fibrillation
